# Transformational Leadership, Employee Engagement, Job Satisfaction, and Psychological Well-Being among Hotel Employees after the Height of the COVID-19 Pandemic: A Serial Mediation Model

**DOI:** 10.3390/ijerph20043609

**Published:** 2023-02-17

**Authors:** Magdy Sayed Ahmed Abolnasser, Ahmed Hassan Abdou, Thowayeb H. Hassan, Amany E. Salem

**Affiliations:** 1 Social Studies Department, College of Arts, King Faisal University, Al-Ahsa 31982, Saudi Arabia; 2 Department of Geography, Faculty of Arts, Ain Shams University, Cairo 11566, Egypt; 3 Hotel Studies Department, Faculty of Tourism and Hotels, Mansoura University, Mansoura 35516, Egypt; 4 Tourism Studies Department, Faculty of Tourism and Hotel Management, Helwan University, Cairo 12612, Egypt

**Keywords:** transformational leadership, engagement, well-being, employee satisfaction, COVID-19, hotel industry

## Abstract

Over the past few years, great attention has been given to the impacts of the COVID-19 pandemic and its consequences on employee psychological well-being (PWB), particularly in the hospitality industry. Like many aspects of human life, employee PWB is influenced by multiple factors. One of the factors that may affect employee PWB is transformational leadership (TLS). Accordingly, we aim through this study to empirically (1) examine the direct effect of transformational leadership on employee PWB and (2) investigate the potential independent and serial mediation effects of employee engagement (EEG) and job satisfaction (JS) on the TLS-PWB relationship after the height of the COVID-19 pandemic. Data were gathered using an online questionnaire from a convenience sample of 403 front-line employees from five-star hotels in Saudi Arabia. The partial least squares structural equation modeling (PLS-SEM) with the bootstrapping technique was utilized to test the study hypotheses. Based on the demands–resources (JD-R) theory, the findings of this study reveal a significant positive effect of TLS on hotel employees’ PWB. Additionally, drawing on the stimulus–organism–response (S-O-R) model, the two main contributions of this study are: (1) EEG and JS serially and independently have a significant partial mediational effect on the TLS-PWB relationship among hotel employees, and (2) EEG has a greater impact on the TLS-PWB relationship as an intervening variable than the two other mediators (JS, as well as EEG and JS serially). Based on these findings, hotel management should mainly consider developing and encouraging TLS behavior among their managers to promote EEG and increase JS among their followers, which consequently enhances their PWB and alleviates negative psychological outcomes due to experiencing a disaster such as the COVID-19 pandemic.

## 1. Introduction

Several industries worldwide have experienced an unexpected, temporary, and sharp decline in revenue due to the COVID-19 pandemic [1]. During the height of the pandemic, the hospitality industry was one of the most severely affected sectors [2,3]. In addition to the physical, psychological, and financial concerns resulting from the COVID-19 pandemic, hospitality workers are also faced with high job demands; long, non-sociable, and irregular working hours; a shortage of workers; inadequate time to spend with family; and poor quality of life [4,5]. Such concerns and working conditions result in psychiatric issues such as anxiety and depression. Therefore, finding mechanisms that contribute to promoting employees’ well-being is crucial [6]. Employee well-being determines employee performance in the workplace. For example, when employees are happy in the workplace, their performance will increase. On the other hand, if an employee is not happy, stressed, or frustrated, their level of performance is likely to be poor [7]. One aspect of employee well-being is psychological well-being (PWB). PWB is a state of contentment, happiness, life satisfaction, and a feeling of accomplishment. It means that if one is happy, contented, satisfied with life, and feels that they accomplished what they set out to achieve, they can be psychologically satisfied [8,9].

Like many aspects of human life, employees’ psychological well-being (PWB) is influenced by multiple factors. Some of these factors are limited to the individual, while others are found within the environment one operates [10,11,12]. One of the important factors that may influence psychological well-being, especially in the workplace, is leadership style [13]. Leadership is key to an organization’s performance, sustainability, and success in the market [14]. In leadership, leaders guide employees on what to do and how to behave and make decisions that determine the direction of a company [15]. In the hospitality industry context, there are different leadership styles, including democratic, autocratic, strategic, transactional, and bureaucratic leadership [16]. Each style has unique characteristics, advantages, and disadvantages. One of the most common leadership approaches that has attracted significant attention from hospitality academic researchers in recent years is transformational leadership (TLS) [17].

In TLS, leaders are responsible for broadening and elevating their followers’ interests, raising their awareness and commitment to the group’s mission and purpose, and enabling them to transcend their interests to benefit the group as a whole [18,19]. Transformational leaders inspire creativity and innovation and increase employees’ interest in their work by employing a visionary and creative leadership style [20,21]. As mentioned in earlier studies, PWB is significantly positively influenced by TLS directly and indirectly [22,23,24,25]. For instance, the systematic review carried out by Arnold [23] to investigate the relationship between TLS and PWB in the period between 1980 to 2015 concluded that, generally, TLS has a significant positive effect on positive measurements of PWB while negatively contributing to predicting the negative measurements of PWB.

In addition to PWB, there is substantial evidence indicating the substantial impacts of TLS on both job satisfaction (JS) and employee engagement (EEG). These effects are closely interrelated in the sense that TSL plays a significant positive role in enhancing JS [24,26] and promoting EEG [27]. Additionally, further empirical investigations illustrated that JS, and EEG, have a significant impact on workers’ PWB. For instance, some scholars [28,29,30] have concluded that JS is regarded as one of the key predictors of employee PWB. This finding suggests that employees satisfied with their careers are more likely to be psychologically healthy. Similarly, with regard to EEG, earlier studies emphasized the vital role of EEG in enhancing employee PWB [31,32]. 

Even though numerous scholars have investigated the link between TLS, JS, EEG, and PWB in different contexts, the link between these variables in the setting of the hospitality sector is still limited, particularly after the height of the COVID-19 pandemic. Further, based on the best knowledge of the authors, the indirect nexus between TSL and PWB in the existence of EEG and JS as mediators has not been examined before. Additionally, no previous empirical studies have examined the potential serial mediation effect of EEG and JS in the TLS-PWB relationship in the context of the hospitality industry or any other context. Consequently, to narrow these gaps in the hospitality sector setting, this study aims to (1) examine the direct effect of TLS on EEG, JS, and PWB, (2) illustrate the direct effect of EEG and JS on employees’ PWB, and (3) explore the potential independent and serial mediation effects of EEG and JS in the TLS-PWB relationship. To achieve these objectives, the following questions are addressed: (1) To what extent does TLS affect the EGG, JS, and PWB of Saudi Arabian hotel employees? (2) To what extent do the EGG and JS affect the PWB of hotel employees? (3) How does EEG influence the TSL-PWB relationship? (4) How does JS mediate the TLS-PWB relationship? (5) How do EEG and JS serially mediate the effect of TLS on PWB among Saudi Arabian hotel employees? 

In comparison to the previous literature on TSL, EEG, JS, and employee PWB, this study contributes to the enrichment of the literature of the hospitality industry context in different noteworthy ways. Firstly, it is the first empirical one that investigates the direct impact of TLS on employee PWB in the hotel industry context after the height of the COVID-19 pandemic. Further, examining the indirect TLS-PWB relationship via EEG and JS as intervening variables is the second significant contribution of this study. Thirdly, this is the first empirical investigation that examines the potential serial mediation effect of EEG and JS in the association with TLS and PWB in the hotel industry context. Fourthly, the developed serial mediation model includes (TLS, EEG, and JS) as independent variables and (PWB) as a dependent one may serve as a valuable foundation for upcoming research in other hospitality sectors.

This study focuses on the hotel sector in Saudi Arabia because of its unique organizational culture which, like any other sector, is deeply rooted in tradition and values. Generally, this organizational culture is characterized by strong hierarchical structures, collective decision-making, and respect for authority [33]. Further, religious beliefs play an important role in the organizational culture of Saudi Arabia. Islamic principles shape the way people think about work, money, and success. Employees often adhere to traditional Islamic values, such as modesty and obedience to rules. Communication styles tend to be formal and indirect, with decisions made based on consensus rather than individual preference. There is also a strong focus on loyalty and respect for seniority in the workplace [33]. 

## 2. An Overview of the Theoretical Background and the Development of Hypotheses

### 2.1. The Concept of Transformational Leadership 

Leadership style, particularly in the setting of the hospitality industry, is considered one of the most effective management tools. If it is used appropriately, it can improve the relationship between managers and their employees, create a more productive work environment, and boost the quality of services provided [34,35]. The success of a hospitality firm depends on motivating its employees to do what they are capable of and reach their full potential, become engaged, adapt to change, and choose technical solutions wisely [35]. The role of an effective leader is to provide guidance that motivates their employees to carry out tasks towards their responsibilities, solve business problems creatively and innovatively, and make and take decisions that benefit the team and company as a whole [36]. All of these characteristics can be found in the transformational leadership style (TLS) [37,38,39].

Recently, in hospitality academic research, TLS is considered to be the most commonly used leadership approach [17]. TLS was initially introduced by James MacGregor Burns, who used it to describe how followers’ values can be influenced and changed by their leaders. According to Bass and Avolio [18], TLS involves changing followers’ perceptions of what is important and inspiring them to see themselves and their environment, as well as its opportunities and challenges, differently. Further, Allen et al. [40] illustrated that TLS occurs when leaders as well as their followers encourage each other to achieve advanced levels of motivation and morality. Avolio et al. [39] and Bass [41] identified the characteristics of transformational leaders and classified them into four categories/dimensions as follows, (1) charisma (idealized influence), (2) “inspiration” (inspirational motivation), (3) “intellectual stimulation”, and (4) “individualized consideration”. Others classified them into five including “vision”, “inspirational communication”, “intellectual stimulation”, “supportive leadership”, and “personal recognition” [42]. The idealized influence of leaders occurs when they choose to act ethically rather than expediently, commit to their followers morally, and put the organization’s interests ahead of their self-interest. The charismatic leader inspires pride, gains respect, demonstrates vision, and builds a sense of mission. Further, inspirational motivation is related to the leader’s ability to communicate and provide clear instructions and expectations, emphasize important points, and simply describe significant goals. To stimulate an individual’s intellectual development, leaders must encourage others to think creatively and explore new ways of accomplishing tasks. In other words, a transformative leader boosts intelligence, enhances rational thinking, and promotes problem-solving skills. Lastly, individualized consideration is based on a leader who deals with their followers as individuals, takes the time to develop his or her employees’ skills, coaches, and advises them. 

During the era of the height of the COVID-19 pandemic, TSL had a considerable positive effect on a wide range of employee/work outcomes in various settings. For example, in the healthcare sector setting, employee performance was significantly improved by TLS, which resulted in a decrease in turnover intentions [43]. Further, one of the positive outcomes found by Charoensukmongkol and Puyod [44] was that TSL significantly improved the balance of work–life and lowered role ambiguity between university employees in the Philippines. In addition, in Jordanian public sector organizations as well as the Indonesian banking sector, TSL significantly contributed to enhancing job satisfaction among HR and bank employees, respectively [45,46]. Furthermore, in Saudi Arabian hotels, Sobaih et al. [47] stated that TLS had a substantial positive effect on psychological safety, which in turn negatively influenced turnover intention among hotel employees. In another empirical investigation conducted by Yuan et al. [48] on 580 hospitality and tourism employees, the results indicate that TSL was a significant and key determinant of employee commitment. In the restaurant industry context, employee commitment to change as well as quality of work–life was significantly increased by TLS [49]. Despite the various negative impacts of the COVID-19 pandemic on the hospitality sector and its employees, TLS is still an effective leadership style that significantly mitigates these adverse outcomes. 

### 2.2. The Concept of Psychological Well-Being

The concept of well-being is a major conceptual psychological framework that supports the development of strengths and resources. When we talk about well-being, different concepts are brought to mind, such as satisfaction with life, happiness, purposefulness, vitality, personal acceptance, and effective functioning [8]. Ryan and Deci [50] classified well-being into two categories including hedonic and eudaemonic well-being. Hedonic well-being (subjective well-being) is commonly referred to as a subjective feeling of pleasure as well as positive emotions. Basically, it consists of the presence of a positive mood, a lack of negativity, and a feeling of fulfillment in one’s life, as well as satisfaction with various domains of life [51]. It is postulated that happiness is considered to occur when both positive affect and life satisfaction are high [8]. However, eudaemonic well-being is usually regarded as psychological well-being (PWB), which was defined by Cowen [52] (p. 404) as “having a sense of control over one’s fate, a feeling of purpose and belongingness, and a basic satisfaction with oneself and one’s existence”. 

An extensive literature review by Ryff and Keyes [9] (p. 727) identified six dimensions of psychological well-being: (1) “self-acceptance”: people who are self-acceptant accept and acknowledge their good and bad qualities as well as feel positive about their past and possess a positive attitude towards themselves. (2) “Positive relationships with others” refers to those people who are capable of developing trusting, warm, and satisfying relationships with others; caring for others’ well-being; being empathetic, affectionate, and intimate; and understanding the challenges and possibilities of life. (3) “Autonomy” refers to being free from social pressure to think or act a certain way, regulating one’s behavior from within, and evaluating oneself according to one’s own standards. (4) “Environmental mastery” means having the ability to manage one’s environment effectively, take advantage of surrounding opportunities, and be capable of selecting a context that is appropriate for one’s needs and values. (5) “Personal growth” implies that the person feels they are growing and expanding, develops themself continually, seeks out new experiences, realizes their potential, and changes to reflect increased self-awareness. (6) “Purpose in life” indicates that the person is goal-oriented and has a sense of direction in life, considers past and present lives meaningful, and strives to accomplish goals and objectives.

During the height of the COVID-19 pandemic, great attention was given to the impacts of COVID-19 and its consequences on employee PWB in various contexts [11,53,54,55,56,57]. For instance, Holton et al. [54] found that the COVID-19 pandemic significantly influenced psychological well-being, especially among nurses and midwives, where anxiety levels were increased. Further, in the hotel industry context, COVID-19 and its related concerns (physical, psychological, financial, and those related to social gaze) significantly affected work stress, which in turn negatively influenced employee well-being and mental health [53]. Another study carried out by Sarwar et al. [57] on a sample of hotel delivery personnel revealed that employee PWB is significantly negatively impacted by job insecurity resulting from COVID-19. Conversely, some scholars have examined the factors that affect the mitigation of the impacts of the COVID-19 pandemic on employee PWB. For instance, Pakenham et al. [58] concluded that psychological flexibility significantly contributed to mitigating the adverse effects of COVID-19 on Italian citizens’ mental health. 

### 2.3. The Relationship between TLS and PWB

Over the past few years, great attention has been given by scholars to the direct relationship between TLS and PWB in several contexts. A review study carried out by Tongtong and Yusuf [59] revealed that TLS plays a significant contribution in enhancing the PWB of subordinates. Further, a highly significant correlation between TLS and PWB (β = 0.687, *p* < 0.001) was mentioned by Widanti and Sunaryo [60] in their study of a sample of 235 participants in some of Surakarta’s hospitals (Indonesia). Moreover, in the hotel industry setting, Kloutsiniotis et al. [61] illustrate that TSL plays a vital role in reducing stress, anxiety, and workplace loneliness caused by COVID-19 and eliminates the adverse impacts of stressors on hotel staff burnout. Sivanathan et al. [62] suggested that TLS’s four dimensions are highly relevant to employees’ PWB. For instance, in times of crisis, leaders who demonstrate idealized influence can shift their focus from short-term financial results to their employees’ long-term health and well-being. Further, an inspirational and motivational leader is able to inspire their followers to overcome psychological hurdles and face future challenges with strength. In addition, Kara et al. [35] confirmed that TLS has a highly significant predictive effect on employees’ quality of life, indicating that TLS significantly increased satisfaction levels of different needs (i.e., health and safety needs; social, economic, and family needs; esteem needs…etc.). Theoretically, in addition to the previous results, the job demands–resources (JD-R) theory could foster the relationship between TLS and PWB. This theory proposes that high job demands combined with low job resources can lead to negative outcomes such as decreased psychological well-being [62,63]. Based on the JD-R theory, TLS can have a positive effect on employees’ psychological well-being. This is because transformational leaders create an environment that provides employees with resources (such as autonomy, sharing in decision-making, providing support, showing respect for individual differences, and recognition) that helps reduce feelings of stress and burnout and can help improve the overall psychological well-being of employees [25,64]. From the previous results, we suggest that TSL can be considered one of the key predictors of employees’ PWB. As a result, the present study will test the following hypothesis:

**Hypothesis** **1** **(H1).** *TLS has a significant positive effect on PWB among hotel employees after the height of the COVID-19 pandemic.*

### 2.4. The Mediating Effect of EEG in the TLS-PWB Relationship 

EEG is one of the most important elements of an effective organization [65]. This is primarily due to the fact that engaged employees are more productive, safer, and healthier than those who are not engaged [66]. According to Schaufeli and Bakker [67], work engagement is regarded as a temporary, positive, satisfying work-related state of mind that is distinguished by vigor, dedication, and absorption. Vigor, in this context, describes the engaged employee who has a high level of energy and mental resilience. Further, dedicated employees are those who are enthusiastic about their work and inspired by their duties. Meanwhile, absorption, in the work engagement setting, refers to being completely focused on the work at hand and feeling like time is flying by [27,67].

In terms of the TLS-EEG relationship, numerous scholars have explored the impact of TLS on EEG in various contexts. For instance, an empirical investigation carried out by Breevaart et al. [68] on a sample of 61 naval cadets found that there was a higher level of engagement among the investigated cadets on days when their leader demonstrated more TLS. Further, in the mining industry context, Bezuidenhout and Schultz [69] concluded that TSL and EEG are significantly positively correlated and should be viewed holistically as a whole. Amid the height of the COVID-19 pandemic, the results of a recent study adopted by Santoso et al. [70] indicate that TLS played a key role in strengthening EEG via internal communication, which in turn alleviated the concerns of employees regarding the COVID-19 pandemic. Similarly, another study aimed at determining to what extent TLS engages the workplace setting among Pakistani bankers during the height of the COVID-19 pandemic suggested that the workplace is more likely to be engaged by leaders who exhibit highly transformative leadership behaviors [71]. Additionally, transformational leadership theory suggests that transformational leaders can create a culture of engagement by instilling trust, providing meaningful work, providing resources to help employees stay focused, and recognizing employees’ contributions [72]. Furthermore, transformational leaders focus on developing employees’ skills and helping them reach their full potential, which can motivate them to work harder and become more engaged [73]. From the previous, it can be assumed that TLS significantly contributes to improving EEG in the context of the hotel sector after the height of the COVID-19 pandemic.

Regarding the EEG-PWB relationship, several studies have found a positive link between EEG and PWB. One of these studies that explicitly examined the correlation between EEG and PWB is that of Shuck and Reio Jr [74]. The results of their ANOVA analysis revealed that the group of employees that demonstrated a higher engagement also exhibited higher psychological well-being than those with a low engagement level. In the hospitality industry context, the findings of another empirical investigation demonstrated that EEG is directly related to psychological well-being. In other words, highly engaged employees may be more psychological well than others who are not [32]. Robertson et al. [75] indicated that, in addition to experiencing positive emotions, engaged employees often experience better physical and psychological well-being. Additionally, in the healthcare sector context during the COVID-19 pandemic, Gómez-Salgado et al. [76] concluded that healthcare professionals who did not declare psychological distress had higher levels of work engagement in all dimensions. They confirmed that improving healthcare professionals’ engagement in their work could prevent them from the psychological distress associated with COVID-19. As a result of what has been stated, we hypothesize that EEG has a significant contribution to promoting employee PWB in the hotel industry context amid the COVID-19 pandemic.

Based on the extensive literature reviewed that examined the interrelationship between TLS and employees’ PWB, it could be mentioned that there are no previous studies that addressed the indirect relationship between these constructs in the existence of EEG as an intervening variable. As a consequence, in order to examine the mediating role of EEG in the TLS-PWB relationship, this study’s theoretical framework will be based on the stimulus–organism–response (S-O-R) model. This model suggests that people’s behavior (R) is determined by both their external environment (S) and their internal reactions (O) [77]. In this model, a stimulus is an object or event in the environment that elicits or triggers a behavior or response, whereas the organism is the individual who responds to the stimulus [78]. It appears that behavior is the result of a combination of factors, such as the stimulus and the individual’s current state, motivations, experiences, and personality. [79]. Using this model, we suggest that TLS (stimuli) could significantly affect EEG (organism), which in turn may significantly contribute to enhancing employees’ PWB (response). Building on what was mentioned above, which reflects the significant positive impact of transformational leadership on EEG and the significant positive contribution of EEG to enhancing PWB, it could be postulated that the higher the TLS perceived, the better the EEG achieved, which in turn could significantly improve the PWB of hotel employees after the height of the COVID-19 pandemic. Consequently, we suggest the following hypotheses:

**Hypothesis** **2** **(H2).** *TLS significantly contributes to improving EEG among hotel employees after the height of the COVID-19 pandemic.*

**Hypothesis** **3** **(H3).** *EEG significantly contributes to promoting PWB among hotel employees after the height of the COVID-19 pandemic.*

**Hypothesis** **4** **(H4).** *EEG significantly positively mediates the TLS-PWB relationship among hotel employees after the height of the COVID-19 pandemic.*

### 2.5. The Mediating Effect of JS in the Relationship between TLS and PWB

In industrial and organizational psychology, it is widely recognized that job satisfaction is one of the most-studied factors [80]. Both academics and practitioners understand the significant role of JS in the prediction of critical organizational outcomes [81]. This is what is referred to as the individual’s feelings and attitude towards their job. Job satisfaction is evidenced by favorable attitudes toward the job. On the contrary, negative and unfavorable attitudes reflect job dissatisfaction [82]. According to Aziri [83], a worker’s job satisfaction refers to the feelings of accomplishment and success they experience at work. Generally, JS is believed to be directly related to personal productivity and well-being. Numerous studies have investigated the factors affecting employee job satisfaction. One of these factors that significantly contributes to improving job satisfaction is transformational leadership.

The relationship between TLS and JS has been extensively researched by various scholars in different settings. There is significant evidence in existing research that TLS improves JS [84,85,86,87]. For example, in the context of the food industry in Bosnia and Herzegovina, results show that when the TLS style is practiced, its four dimensions significantly contribute to enhancing JS [87]. Further, the findings of a systematic review study applied to demonstrate the influence of TLS on hospital staff JS revealed that TLS has a great effect on JS among hospital staff and recommended that in order to increase JS among medical staff, practicing TLS is essential [84]. Additionally, in the Ghanaian tourism and hospitality sector, employee JS was significantly increased by TLS (β = 0.362, *t*-value = 3.956, *p* < 0.001) [86]. In the era of COVID-19, TLS plays a significant role in enhancing JS. An empirical study aimed at examining the influence of TLS on governmental employees’ JS amid COVID-19 in Jordon emphasized that the five dimensions of TLS, namely, “deal with uncertainty”, “guidance and support”, “support teamwork”, “effective communication”, and “risk management” significantly have a strong positive impact on JS [44]. In addition to the previous findings, the theory of transformational leadership, which explains the unique connection between a leader and their followers, postulates that a leader’s ability to foster creativity, create a positive work environment, and promote open communication could all play a significant role in job satisfaction [88]. From these findings, we can assume that the greater the perceived TLS, the higher the employees’ JS. 

In the context of the JS-PWB relationship, some studies have examined this relationship [28,29,30]. The findings of these studies confirmed the significant positive effect of JS on the PWB of employees. It is noted that the majority of these studies were applied in the healthcare sector. For instance, Park et al. [89] in their empirical investigation carried out on a sample of Malaysian mental health practitioners illustrated that JS is significantly associated with PWB, which was measured by lower levels of perceived stress (β = −0.51, *p* < 0.001) and less mental health issues (β = −0.33, *p* < 0.01). In other words, the higher the perceived JS, the lower the perceived stress and the fewer mental health issues. Another study was conducted to examine the influence of JS on PWB in a sample of 110 psychiatric nurses in Nigeria, stating that nurses’ PWB is significantly affected by JS, implying that when nurses are satisfied with their occupation, they are probable to have a higher level of PWB [28]. Furthermore, the findings of Nielsen et al. [24] show that JS is significantly positively correlated with the PWB of elderly care employees (β = 0.38, *p* < 0.01). 

Similarly, as in employee engagement, there is a paucity of empirical studies that have researched JS as a mediator between TLS and PWB. As a result, drawing on the S-O-R model, we suggest that TLS (stimuli) could significantly affect JS (organism), which in turn may significantly contribute to promoting employees’ PWB (response). Based on the results of previous studies, which evidence that TLS, JS, and PWB are significantly positively correlated, it can be assumed that the practice of TLS within the work environment may lead to an increase in employees’ JS which, accordingly, may result in better employees psychological well-being. Upon that, we hypothesize that:

**Hypothesis** **5** **(H5).** *TLS significantly contributes to increasing JS among hotel employees after the height of the COVID-19 pandemic.*

**Hypothesis** **6** **(H6).** *JS significantly contributes to promoting PWB among hotel employees after the height of the COVID-19 pandemic.*

**Hypothesis** **7** **(H7).** *JS significantly positively mediates the TLS-PWB relationship among hotel employees after the height of the COVID-19 pandemic.*

### 2.6. The Impact of EEG on JS

In the context of the EEG-JS relationship, numerous studies that extensively examined this causal relationship indicate that EEG has a significant positive effect on JS [90,91,92,93,94]. An empirical study carried out on a sample of 594 employees from the Savinja Statistical Region in Slovenia found that EEG has a significant positive influence on JS (β = 0.545, *p* < 0.05) [90]. Likewise, the findings of the review study conducted by Bin Shmailan [92] illustrate that EEG and JS are significantly correlated. In the IT sector context, the results of regression analysis indicate that JS is substantially affected by EEG (β = 0.532, *p* < 0.001) [95]. Moreover, in the private bank sector setting, Madan and Srivastava [96] concluded that EEG significantly contributes to improving bankers’ JS. Similarly, another empirical investigation adopted on a sample of 121 employees in an Indonesian chemical manufacturing company stated that work engagement significantly increased employees’ JS, suggesting that the higher the perceived work engagement, the greater the JS [97]. Furthermore, drawing on social exchange theory, EEG can significantly affect JS. According to this theory, employees engage with their work because they receive some benefit from it (such as recognition or rewards) [98,99]. Upon that, if employees are engaged in their work and feel appreciated by their employer through a social exchange of rewards, they may be more likely to be satisfied with their job. Conversely, if they are disengaged from their work or do not feel appreciated by their employer through a social exchange of rewards, they may be more likely to be dissatisfied with their job [100]. From the previous results, it can be postulated that:

**Hypothesis** **8** **(H8).** *EEG has a significant positive effect on JS among hotel employees after the height of the COVID-19 pandemic.*

### 2.7. Serial Mediation Effect of EEG and JS on TLS-PWB Relationship

Earlier studies have argued that transformational leaders provide an appropriate work environment for their followers. They convey the organization’s vision, mission, and values, as well as encourage them to solve business problems creatively and innovatively, in addition to acting as role models [33,34,35]. Employing these practices in the work environment significantly contributes to improving employee engagement, which eventually leads to enhancing and increasing the level of employee job satisfaction [65,67,68,81,82]. Both EEG and JS substantially positively affect employee PWB. In addition, based on the S-O-R model, we suggest that TLS (stimuli) could significantly affect EEG and JS serially (organism), which in turn may significantly contribute to promoting employees’ PWB (response). More specifically, the employees who perceive they have high levels of TLS will have a higher level of EEG, which then may directly increase their job satisfaction. Accordingly, EGG and JS serially may promote PWB (such as feeling positive towards present and past lives and the ability to develop warm, fulfilling relationships with others and having goals and objectives in life) among hotel employees particularly after the height of the COVID-19 pandemic. Hence, we suggest that:

**Hypothesis** **9** **(H9).** *EEG and JS serially have a significant positive mediating effect on the TLS-PWB relationship among hotel employees after the height of the COVID-19 pandemic.*

**Figure 1:**
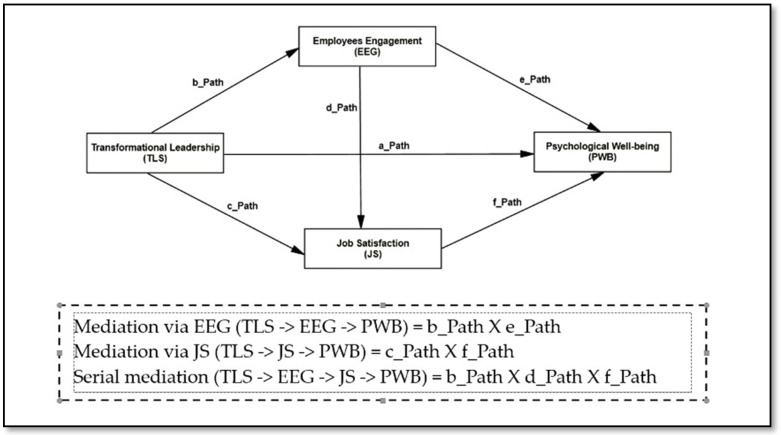
Study conceptual framework.

This study’s conceptual framework is illustrated in Figure 1. 

## 3. Materials and Methods

### 3.1. Measures and Instrument Development

As a part of this study, primary data collection was gathered via a web-based questionnaire survey. Especially in quantitative research methodology, online questionnaires have become increasingly popular as a means of data collection over the internet [101]. A comprehensive review of the literature was conducted in order to detect valid and frequently used measures for developing the study’s questionnaire. This questionnaire was divided into five parts. Participant demographic characteristics are gathered in the first part, which includes age, gender, educational level, current position, and working experience in the investigated hotel. Part two presents how the investigated respondents perceive transformational leaders’ behaviors. The third and fourth parts show how the investigated employees are engaged and satisfied with their jobs, respectively. In the last part, we consider measuring the level of PWB among the investigated participants.

A seven-item scale developed by Carless et al. [102] for measuring TLS was adapted and utilized. Samples of these items are “your supervisor/manager gives recognition and encouragement to staff; your supervisor/manager treats employees as individuals, supports and encourages their development.” Based on a Likert scale of 1 to 5, where 1 = “rarely or never”, and 5 = “very frequently, if not always”, participants were asked to rate how often their managers are involved in the described behavior. The greater the mean score, the higher the perceived transformational leadership. The TLS’s internal consistency was excellent (α = 0.925).

Measurement items for PWB were based on those developed and validated by Pradhan and Hati [103]. The PWB of the investigated participants was assessed based on a modified ten-item scale. An example of these items is “I am a confident person”. According to the internal consistency reliability test, Cronbach’s alpha was extremely high (α = 0.924). With regard to EEG, a nine-item scale developed by Thomas [104] was employed to measure how the investigated participants are engaged with their jobs. The following is a sample of the items on this scale: “your job is a source of personal pride”. This scale indicated excellent internal consistency (α = 0.942). Lastly, JS was measured using a 5-item scale “Andrews and Withey job satisfaction scale” validated by Rentsch and Steel [105]. These items are focused on measuring job satisfaction, the work itself, co-workers, the work conditions, and the availability of resources to do the job. The scale shows good internal consistency (α = 0.879). The higher mean indicates a higher level of JS. PWB and EEG were generally rated by the investigated participants using a 5-point Likert scale, 1 = strongly disagree, 5 = strongly agree. Meanwhile, JS was rated on a 5-point scale, 1 being strongly dissatisfied and 5 being highly satisfied. In Appendix A, we present the study’s constructs and their associated items. 

The questionnaire form was originally developed in English. Two researchers fluent in both Arabic and English translated it from English into the native Arabic language of the participants. A back-translation was performed by two other experts after the Arabic translation was completed in order to ensure that there were not any linguistic differences between the Arabic and English versions. Both the original and the revised translated version were identical. For this study, content validity was crucial for the survey questionnaire. As a means of ensuring that the questionnaire’s content validity was accurate, three hospitality scholars reviewed the survey content and provided feedback. Additionally, a pilot study was conducted to confirm the questionnaire’s consistency, simplicity, and clarity as well as identify any ambiguities between terms and meanings. The study was adopted on twenty-five participants who were not included in the main sample. In response to feedback from participants and scholars, modifications were made to some questionnaire statements. Moreover, the order of some statements was also rearranged.

### 3.2. Sample of the Study and Data Collection

As illustrated previously, the main aim of this study is to empirically examine the direct impact of TLS on EEG, JS, and PWB, and further to demonstrate the direct impact of EEG and JS on employees’ PWB, as well as explore the potential independent and serial mediating effects of EEG and JS in the TLS-PWB relationship in a sample of five-star Saudi Arabian hotels after the height of the COVID-19 pandemic. To achieve this aim and its associated objectives, a convenience sample of employees from five-star hotels was surveyed using an online questionnaire. In convenience sampling, a sample is selected from individuals who are easily reached or contacted. It is the most commonly used sample that has different advantages such as saving time and money, does not require listing all of the elements of the population, participants are easy to reach, and data are collected quickly [106]. 

The study targeted frontline employees who have direct contact with hotel guests. The study focused on employees from five-star hotels. Based on https://www.booking.com/ (accessed on 4 April 2022), 147 hotels were identified [107]. Most of the five-star hotels were located in Riyad, Jeddah, and Makkah cities. The questionnaire was designed and uploaded on the Google form platform. Human resource managers in these hotels were contacted for us to be permitted to distribute the questionnaire among their employees. After obtaining approval, participants who had agreed to participate were asked to sign a consent form and then were provided with a form link that they could use to access the questionnaire to respond. In addition to a welcome message, a detailed description of the study’s objectives was identified. Participants in this study were informed that participation was voluntary. Finally, they were kindly informed that the survey had to be resubmitted once they completed it. Almost two months (May–July 2022) were spent gathering data. In total, 420 forms were received, of which only 403 were valid for statistical analysis. 

Using the suggestions of Nunnally [108], a suitable sample size was identified. According to him, the sample size should be determined by the number of items examined in the study. Maintaining a ratio of 1 to 10 is good (item: sample). As a result, 31 items would require the participation of 310 respondents. Our sample size of 403 participants in this study was adequate. This is also in accordance with Hair et al.’s [109] recommendation to use 100 to 150 samples for maximum likelihood estimation (MLE). Additionally, it is consistent with Boomsma’s recommendation that structural equation modeling uses a minimum of 200 samples [110]. Table 1 shows the demographic characteristics of the respondents.

### 3.3. Data Analysis

In the current study, the analysis of data was conducted with SPSS v. 22 and SmartPLS v. 4.0.8.4. The demographics of the investigated participants as well as their opinions about the study’s variables were represented by descriptive statistics. We tested the reliability and validity of the measurement items using confirmatory factor analysis (CFA) and Cronbach’s alpha. Detecting common method variance (CMV) was done by applying the Harman single-factor test. To check the study’s convergence validity, composite reliability (CR) was calculated along with average variance extracted (AVE). Furthermore, in addition to the Fornell–Larcker criterion, cross-loading of indicators was employed to assess discriminant validity. The structural model fit, quality, predictive accuracy, and predictive relevance were evaluated by calculating the coefficient of determination (R^2^), Q^2^ predict, as well as the predictors’ effect size (f^2^). Finally, the partial least squares structural equation modeling (PLS-SEM) with bootstrapping technique was utilized to test the study hypotheses and determine the statistical significance of the study results. 

## 4. Results

### 4.1. Analysis of the Respondents’ Demographic Characteristics

As mentioned previously, a total of 403 valid responses were obtained. As shown in Table 1, 79.7% of these participants were males. In contrast, 20.3% of the respondents were females. More than half of the investigated participants (50.4%) were between the ages of 20 and 30 years, followed by those between the ages of 31 and 40 years (41.2%). Older participants (ranging from 40–50 years) had the lowest percentage (8.4%). According to their level of education, about two-thirds of the investigated respondents (65.7%) hold a university degree, while 25.1% hold a high school degree, followed by those who hold a postgraduate degree (9.2%). With regard to their departments, a higher percentage (39.2%) work in the food and beverage department, followed by 32.5% working in the front office department. In terms of work experience in the investigated hotels, 46.2% worked for 5–10 years, followed by those working for less than 5 years (44.4%) and more than 10 years (9.4%). 

### 4.2. Common Method Variance (CMV) 

As the data were collected through an online survey, there may be a common method variance/bias. For this reason, three methods, namely anonymity, confidentiality, and honesty, were implemented to reduce the possibility of CMV [111]. All information and responses from research participants remained confidential and anonymous and were only used for the study’s purposes. The likelihood of detecting response bias is reduced when anonymity is assured [112]. Further, we kindly requested that all participants answer all questions honestly. When honesty is assured, response bias is reduced [113]. In addition to the previous, Harman’s single-factor test was employed in order to detect CMV. Using exploratory factor analysis, one factor explained 40.1% of the variance. When one factor explains more than 50% of the variance, CMV may be an issue. Accordingly, CMV posed no significant issue for the current study [114]. 

### 4.3. The Measurement Model Assessment

The PLS-SEM algorithm was employed to evaluate the reliability as well as the validity of the study’s construct. To assess the reliability of the study’s constructs, two methods were utilized: (1) composite reliability (CR) and (2) Cronbach’s α. As shown in Table 2, a high level of CR and Cronbach’s α value were achieved. CR scores ranged from 0.912 to 0.975 and Cronbach’s α value ranged from 0.879 to 0.942. These values exceeded the threshold of 0.70 as suggested by Ref. [109], assuring excellent internal consistency reliability. The validity of the study constructs was analyzed by considering their convergent and discriminant validity. To support convergence validity, the average variance extracted (AVE) for each examined construct should be higher than 0.50 and the outer loadings for each item should be above 0.70 [109]. The results in Table 2 reveal that all outer loading of the study items is significant (*p* > 0.001) and higher than 0.70 (ranging from 0.705 to 0.985). In addition, all study constructs’ AVE scores are greater than 0.50 (ranging from 0.631 to 0.813), indicating that convergent validity is assured. 

A construct’s discriminant validity indicates its empirical differentiation from other constructs. Two pieces of statistical evidence were applied to evaluate the constructs’ discriminant validity. The first method was Fornell and Larcker’s criterion. According to this method, to sustain discriminant validity, Hair et al. [109] suggest that the square root of AVE must exceed the correlation between the construct and the other constructs. Based on the results in Table 3, the construct’s AVE square root exceeded its correlation with other constructs, indicating a good discriminant validity. 

Examining the cross-loadings of the indicators was the second method of verifying discriminant validity. Using this method, each indicator must have a higher cross-loading on its assigned construct than the cross-loading on the other constructs [109]. The results in Table 4 show that all indicators load more on their construct than on others, indicating the establishment of discriminant validity.

### 4.4. Results of Collinearity Analysis

When regression analyses are performed using the inner model, the results’ significance and values can be biased by high correlations between the constructs [109]. As a result, collinearity must be examined before assessing structural relationships to avoid biasing the results of the regression. When the variance inflation factor (VIF) is greater than 5, the predictors are likely to be collinear [109]. Other researchers mentioned that the presence of collinearity problems at VIF values of 3–5 is also possible [115,116]. Accordingly, a VIF value of 3 or less is ideal, as Hair et al. [109] recommended. Results in Table 4 reveal that all VIF values of all constructs’ indicators are less than 3, indicating that collinearity does not cause a significant issue in this study. 

### 4.5. Assessment of the Structural Model Fit 

Ideally, researchers should evaluate the structural model’s quality first and foremost, according to Hair et al. [109]. Quality is determined by how well the model predicts endogenous constructs. To assess the structural model’s quality, several assessment criteria should be taken into consideration, including the coefficient of determination (R^2^) and Q^2^ predict, as well as the predictors’ effect size (f^2^) [109].

The coefficient of determination (R^2^) was used to measure the model’s predictive accuracy. This also determines the impact of the exogenous construct on the endogenous ones. As mentioned by Hair et al. [109], R^2^ values of 0.75, 0.50, and 0.25 can be categorized as substantial, moderate, and weak, respectively. Regarding effect size (f^2^) values, which represent the observed change in R^2^ when removing a particular construct, Cohen [117] classified f^2^ effect sizes as small, medium, and large owing to their values of 0.02, 0.15, and 0.35, respectively. Additionally, Q*^2^* predict was measured to identify the model’s predictive relevance. In SmartPLS 4, the Q^2^ value is determined by the PLSpredict technique instead of blindfolding as in the previous versions. In PLSpredict, the Q^2^ value is used to compare PLS path prediction errors with simple mean predictions. For better predictive relevance, Q^2^ predicts the value of endogenous constructs should be higher than zero [109,118]. 

Results in Table 5 reveal that all R^2^ values of the exogenous constructs on the endogenous ones are higher than 0.50, indicating a good predictive accuracy of the study model. With regard to the f^2^ values, the results reveal that the f^2^ effect size ranged from 0.028 (small) for TLS on PWB to 1.287 (high) for TLS on EEG assuring model adequacy. Finally, the Q^2^ predict values for endogenous variables were higher than zero, confirming the model’s predictive relevance.

### 4.6. Testing the Study Hypotheses

**Figure 2:**
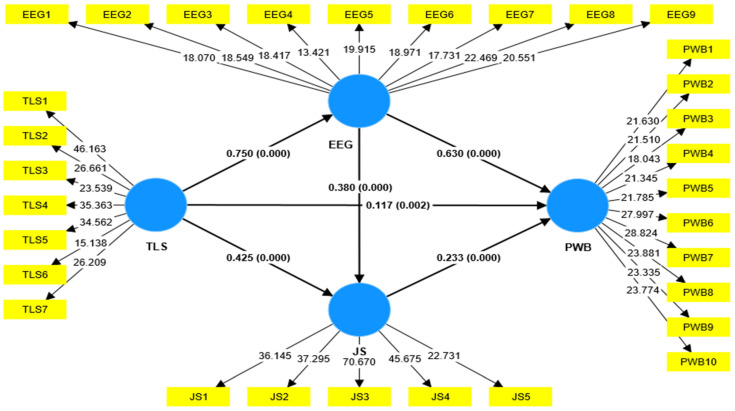
The structural model.

The study hypotheses were tested using PLS-SEM. The path coefficient between independent and dependent variables was statistically tested using bootstrapping. Bootstrapping with subsamples of 5000 was applied. Results from the PLS-SEM are shown in Table 6 and Figure 2.

As shown in Table 6 and Figure 2, the results of PLS-SEM with bootstrapping technique illustrate that TLS significantly positively affects employees’ PWB (β = 0.117, *t*-value = 3.117, *p* < 0.01). As a result, H_1_, which suggests that TLS has a significant positive impact on PWB among hotel employees was supported. Moreover, hypothesis 2, which postulates that TSL significantly contributes to improving *EEG,* is also accepted (β = 0.750, *t*-value = 31.919, *p* < 0.001). Moreover, the results also reveal that EEG *highly* contributes to promoting PWB (β = 0.630, *t*-value = 18.370, *p* < 0.001). Hence, H_3_ is accepted. Similarly, TLS significantly contributes to increasing job satisfaction among hotel employees in the investigated properties. As a result, H_5_ is supported (β = 0.425, *t*-value = 8.116, *p* < 0.001). In terms of the nexus between JS and PWB, our results confirm that JS significantly enhances PWB among hotel employees. Consequently, H_6_ is accepted (β = 0.233, *t*-value = 6.004, *p* < 0.001). Finally, the results also indicate that EEG significantly increases JS (β = 0.380, *t*-value = 7.393, *p* < 0.001). Consequently, H_8_ is supported. 

For the purpose of investigating the indirect TLS-PWB relationship, a bootstrapping technique was employed to examine how EEG and JS may mediate this relationship. The results presented in Table 6 emphasize the significant indirect effect of TLS on PWB through EEG (β = 0.472, *t*-value = 16.561, *p* < 0.001) confirming H*_4_*, which suggests that EEG significantly positively mediates the relationship between TLS and PWB among hotel employees after the height of the COVID-19 pandemic. The bootstrapping analysis also demonstrated that PWB among hotel employees is indirectly (through JS), significantly influenced by TLS (β = 0.099, *t*-value = 4.401, *p* < 0.001), assuring H*_7_*. Regarding the serial mediation effect of EEG and JS on the TLS-PWB relationship, the results confirm that EEG and JS serially significantly mediate the relationship between TLS and PWB, which confirms H_9_. 

In this study, both full and partial mediation suggestions proposed by Kelloway [119] and Zhao et al. [120] were used to examine the effect of the roles played by EEG and JS in mediating the nexus between TLS and PWB. They illustrate that full mediating is only possible when the indirect impact is significant and the direct impact is insignificant. Meanwhile, partial mediating effects could only be detected where both paths (direct and indirect) were significant. As shown in Table 6, the results confirm that EEG and JS independently and serially have a significant partial mediating effect on the TLS-PWB relationship. 

## 5. Discussion

Despite ever-changing and demanding market conditions, Saudi Arabia continues to be a highly competitive environment for the hospitality industry worldwide [121]. The organizational culture of the hotels in Saudi Arabia is often heavily influenced by Islamic values, which are centered on hospitality and respect for all. This culture places a high emphasis on providing excellent customer service, as well as ensuring that all guests feel safe and welcome. Additionally, it also typically involves an element of hierarchy, with top-level management overseeing the operations and making decisions on behalf of the company. This hierarchical structure ensures that employees understand their roles within the organization and take responsibility for their tasks. Furthermore, it allows for clear communication between staff members and management, which is essential in providing effective services to customers [122].

In this study, we aim to empirically examine the direct impact of TLS on EEG, JS, and PWB and, furthermore, demonstrate the direct impact of EEG and JS on employees’ PWB, as well as explore the potential independent and serial mediating effects of EEG and JS on the TLS-PWB relationship in a sample of five-star Saudi Arabian hotels after the height of the COVID-19 pandemic.

This study’s hypotheses tests yield several significant findings, including the following: First, the findings indicate that TLS significantly contributes to promoting the PWB among the investigated hotels’ employees. These findings assure that the greater the perceived TLS, the better the PWB among employees in the hotel industry context. In other words, leadership with transformational characteristics is the key predictor for adapting to day-to-day change and managing responsibilities well, feeling flexible, being capable of decision-making, being free of depression, having direction in life, and being a confident person. By the results of earlier studies, these findings support the close relationship between TLS and PWB [23,24,123]. For instance, Nielsen et al. [24] in the healthcare sector context reported that TLS significantly correlated with the employees’ PWB in a Danish local governmental department. Additionally, amid the COVID-19 pandemic, this study’s findings support those concluded by Irshad et al. [123] that safety-specific TLS had a substantial positive effect on PWB among healthcare workers in Pakistan (β = 0.36, *p* < 0.01).

Second, this study also confirmed that TLS positively significantly affects EEG, suggesting that a higher TLS is more likely to promote EEG among the investigated hotels’ employees. These findings are in line with earlier studies and foster the results of numerous scholars who suggested that leaders with transformational characteristics significantly contribute to making their followers completely devote themselves to their job responsibilities, be able to reach challenging work goals and maintain a high level of performance at their work [65,124,125]. According to Thisera and Sewwandi [125], the four dimensions of TLS significantly improved engagement among executive-level employees in the Sri Lankan hospitality sector context. Among these dimensions, engaging hotel employees was effectively and largely influenced by inspirational motivation and intellectual stimulation. 

Third, concerning the influence of TLS on JS, the findings assure us that TSL plays a vital role in predicting JS, confirming that TLS significantly contributes to increasing employees’ satisfaction with their job, the work they do in their jobs, the availability of resources, the physical surroundings, and supervision. These findings provide further support to the previous findings that illustrate that JS is significantly positively influenced by TSL [24,26,126,127,128]. For example, in the Malaysian hospitality industry setting, Moin et al. [114] found that TLS significantly affected follower job satisfaction. In addition, the empirical investigation carried out by Ohunakin et al. [129] on a sample of 324 employees in university guesthouses in Nigeria revealed that JS was significantly improved by the four dimensions of TLS. Further, among bank sector employees, Dappa et al. [126] stated that TLS is positively significantly associated with JS. Based on the aforementioned findings, it can be suggested that the greater the perceived TLS, the higher the perceived job satisfaction. 

Fourth, regarding the effect of EEG on PWB, the findings resulting from PLS-SEM confirmed that employees’ PWB is highly significantly and positively impacted by EEG, which implies that the higher the employee engagement, the better the employees’ PWB. These results are consistent with those concluded by Kim and Jang [32] that work engagement has a considerable contribution to improving PWB among hospitality employees in the US. In addition, in the software industry context, Joy and Sinosh [31] found that EEG has a substantial effect on PWB, especially on pleasure and purpose dimensions. Additionally, our findings foster the earlier work by Jena et al. [130], confirming that EEG directly has a significant positive impact on PWB.

Fifth, in the context of the JS-PWB relationship, the study’s results emphasize the significant role of JS as a crucial determinant of employee PWB. These findings are in accordance with those mentioned by Emmanuel and Odusanya [28], who found that there is a significant positive relationship between JS and PWB among mental health nurses. These results also support the findings of Chitra and Karunanidhi [30], who illustrated that the PWB of policemen is significantly enhanced once job satisfaction is increased. Furthermore, in a recent study by Alrawadieh et al. [29], PWB was significantly positively impacted by the JS of Turkish female tour guides (β = 0.525, *p* < 0.001). In light of the previous findings, hotel employees are likely more likely to promote PWB when their JS is high.

Sixth, in the context of the relationship between EEG and JS, the findings confirm the significant positive relationship between EEG and JS. These findings are in agreement with the results of the previous works [90,91,92,93], which suggest that EEG has a positive and considerable influence on JS in various settings. For example, in the information technology industry setting, Kamalanabhan et al. [91] confirmed that EEG strongly positively affected JS (β = 0.81, *p* < 0.001). Further, an empirical study carried out on a sample of 200 teachers in the Gwalior region in India illustrated that EEG significantly contributed to increasing JS among the investigated teachers [92]. As a result, it can be suggested that employees’ job satisfaction increases once they have been engaged. 

Seventh, in terms of the intervening role of EEG on the TLS-PWB relationship, this study found that EEG has a significant partial impact on the relationship between both factors. It confirms that employee engagement increases with perceived TLS, which contributes to the promotion of PWB among hotel employees. Therefore, it can be concluded that when employees perceive a high level of transformational leadership, they will be highly engaged in their work, which in turn leads to having a higher level of psychological well-being than those who are not. Similarly, with regard to JB, the study’s findings found that JS significantly partially mediated the nexus between TLS and PWB, suggesting that a highly perceived TLS leads to the increase in JS, which in turn subsidizes PWB among hotel employees. 

In addition to the independent mediation effect of EEG and JS on the link between TLS and PWB, the results of PLS-SEM revealed that EEG and JS serially have a significant partial mediational effect on the TLS-PWB relationship among hotel employees. These findings confirm that hotel managers displaying TLS behavior can enhance employee engagement (i.e., dedication to performing well, being innovative and creative, and offering high-quality products or services with enthusiasm), which consequently results in a high level of job satisfaction. These outcomes (i.e., EGG and JS) serially and significantly contribute to boosting the psychological well-being of hotel employees after the height of the COVID-19 pandemic.

## 6. Theoretical and Practical Implications 

### 6.1. Theoretical Implications

In various ways, the findings of our study substantially contribute to enriching the existing literature review on TSL, EEG, JS, and PWB in the context of the hotel sector specifically after the height of the COVID-19 pandemic as follows. First, the findings illustrate that TLS significantly affects PWB. Based on this finding, the significant positive effect of TLS on PWB can be emphasized. Not only is this the case, but this finding also theoretically supports and extends the usage of job demands–resources (JD-R) theory in the context of the transformational leadership–psychological well-being relationship, which in turn leads to the conclusion that the higher the perceived TLS, the better the PWB achieved. Second, the findings of this study illustrate the significant positive effect of TLS on JS and EEG. From a theoretical point of view, these findings support and extend the application of transformational leadership theory in determining the impact of TLS on JS and EEG. Transformational leadership theory emphasizes the importance of a leader’s ability to motivate and inspire their team, which can lead to increased employee satisfaction and engagement. By demonstrating commitment, setting a good example, and providing clear direction, transformational leaders can foster an environment where employees feel satisfied, valued, and motivated to contribute. This can create a sense of purpose and empowerment that encourages employees to take initiative, be creative, and collaborate with their peers. Additionally, transformational leaders are known for recognizing individual contributions and celebrating successes, which can be an effective way to boost morale, increase satisfaction, and promote engagement. Third, there is a lack of empirical studies that examine the mechanisms by which TSL may affect employees’ PWB. Hence, two mechanisms (EEG and JS) were examined that significantly positively affected the TSL-PWB relationship independently. In other words, based on the best knowledge of the authors, this is the first empirical investigation in the hospitality industry context that demonstrates the roles of EEG and JS as intermediating variables in predicting the TLS-PWB relationship after the height of the COVID-19 pandemic. These findings indicate that the TLS-PWB relationship is significantly partially mediated by EEG and JS. Fourth, no previous studies have been conducted to examine the serial mediation effects of EEG and JS on the TSL-PWB relationship. As a result, this is another substantial contribution of this study, where our findings illustrate that EEG and JS serially significantly partially mediate the relationship between TLS and PWB. Fifth, the main contribution of our study is that employee engagement has a greater impact on the TLS-PWB relationship, as an intervening variable, more than the two other mediators (JS as well as EEG and JS serially). Considering the third, fourth, and fifth contributions, these findings foster and extend the application of the stimulus–organism–response (S-O-R) model in exploring the indirect relationship between TLS and PWB in the existence of JS and EEG as mediating variables. This contributes to the literature on leadership by showing that these two mediating variables have a significant effect on psychological well-being. It also shows that the S-O-R model can be effectively used to study these types of relationships. Sixthly, the social exchange theory was also supported and extended to determine the significant positive effect of EEG on JS. Finally, the developed serial mediation model may be a valuable guide for hospitality scholars in future research to provide an in-depth understanding of direct and indirect paths between TLS and PWB. 

### 6.2. Practical Implications

Some practical implications should be considered by hotel managers and supervisors based on the findings of this study. First, the study findings emphasize the significant role of TLS in predicting and promoting employee PWB directly and indirectly. This study illustrates that TLS significantly promotes PWB by four paths. The first path is that TLS directly promotes PWB. The second and third paths are by EEG and JS independently. The fourth path is through EEG and JS sequentially. Hence, hotel management should mainly consider developing and encouraging TLS behavior among their managers and supervisors to promote EEG and increase JS among their followers, which in turn leads to enhancing their PWB and alleviating the negative psychological outcomes due to experiencing a disaster such as the COVID-19 pandemic. Transformational leaders should motivate followers and promote their positive development; encourage them to think creatively and innovatively; create a culture of high moral standards within the hotel and encourage employees to follow suit; set clear standards, priorities, and values for fostering an ethical work environment; communicate openly; and be authentic and cooperative. Second, this study confirms that employee engagement is the most effective mediator in the relationship between TLS and PWB. In addition, EEG was the most effective predictor among the examined constructs in promoting employee PWB. As a result, the engagement of hotel employees is essential to improve their psychological well-being. It is common for engaged employees to form an emotional connection with their jobs and organizations and strive to achieve organizational goals. Employers could increase EEG in different ways, including establishing clear expectations, rewarding and promoting excellent performance, offering employees regular feedback, informing them about hotel performance regularly, creating a sense of value, and making them feel respected. Third, likewise, regarding job satisfaction, the findings revealed that JS significantly contributes to promoting the employee PWB and positively affects the TLS-PWB relationship. Consequently, ensuring that employees are more satisfied with their jobs, the work they do, their co-workers, the work conditions, and the availability of resources to do the job is essential. The more satisfied employee feels psychologically healthier than the dissatisfied one. 

## 7. Conclusions and Research Limitations

This study aimed to empirically examine the direct effect of TLS on employee PWB and investigate the potential independent and serial mediation effects of EEG and JS on the TLS-PWB relationship after the height of the COVID-19 pandemic. Data were gathered using an online questionnaire from a convenience sample of 403 front-line employees from five-star hotels in Saudi Arabia. The findings of this study reveal a significant positive effect of TLS on hotel employees’ PWB. Additionally, the two main contributions of this study are: (1) EEG and JS serially and independently have a significant partial mediational effect on the TLS-PWB relationship among hotel employees, and (2) EEG has a greater impact on the TLS-PWB relationship as an intervening variable than the two other mediators (JS, as well as EEG and JS serially).

In this study, there are some limitations that should be mentioned. First, the study collected data from a convenience sample of frontline employees at five-star Saudi Arabian hotels, which limits its generalizability. Consequently, the generalizability of this study’s findings needs to be re-evaluated and reconfirmed in other countries, organizational and cultural contexts, and different work settings. Second, the study only examined the potential intermediating roles of EEG and JS in the TSL-PWB relationship independently and serially. Further research may examine other mediators and moderators (i.e., empowerment, work–life balance, social support, resilience, and career adaptability). Third, the demographics of the investigated respondents (i.e., age, educational level, and work experience) may act as moderators in the TSL-PWB relationship that have not been examined in the present study. Forthcoming research may explore the potential moderating effect of these features in this relationship. Fourth, in this study, we utilized TLS as a unidimensional construct without its four dimensions. This is because we as researchers were looking to measure the overall effect of present transformational leadership, rather than the specific aspects that make up the construct. By measuring it as a unidimensional scale, we as researchers were able to focus on the overall level of transformational leadership present in this study and could make more accurate comparisons between different groups or individuals. For forthcoming research, it may be valuable to expand the research to including the four dimensions to demonstrate which one is more predictive in this relationship.

## Figures and Tables

**Table 1:** The respondents’ demographic characteristics.

Characteristic	No.	%
Gender		
Female	82	20.3
Male	321	79.7
Age		
20 to 30 years	203	50.4
31 to 40 years	166	41.2
41 to 50 years	34	8.4
Educational level		
High school degree or equivalent	101	25.1
University degree	265	65.7
Postgraduate degree	37	9.2
Current position		
Front office employees	131	32.5
Food and beverage employees	158	39.2
Housekeeping employees	94	23.3
Others	20	5.0
Working experience in the hotel		
Less than 5 years	179	44.4
5 to 10 years	186	46.2
More than 10 years	38	9.4
Total	403	100%

**Table 2:** The properties of reliability and validity of the study’s constructs.

Construct	Item	Outer Loading	α ^1^	CR ^2^	AVE ^3^
Transformational leadership	TLS1	0.939 ***	0.925	0.953	0.747
	TLS2	0.895 ***			
	TLS3	0.835 ***			
	TLS4	0.923 ***			
	TLS5	0.910 ***			
	TLS6	0.706 ***			
	TLS7	0.817 ***			
Employee engagement	EEG1	0.858 ***	0.942	0.975	0.813
	EEG2	0.940 ***			
	EEG3	0.911 ***			
	EEG4	0.705 ***			
	EEG5	0.960 ***			
	EEG6	0.952 ***			
	EEG7	0.805 ***			
	EEG8	0.985 ***			
	EEG9	0.960 ***			
Job satisfaction	JS1	0.807 ***	0.879	0.912	0.675
	JS2	0.797 ***			
	JS3	0.882 ***			
	JS4	0.855 ***			
	JS5	0.761 ***			
Psychological wellbeing	PWB1	0.777 ***	0.924	0.945	0.631
	PWB2	0.722 ***			
	PWB3	0.753 ***			
	PWB4	0.799 ***			
	PWB5	0.827 ***			
	PWB6	0.846 ***			
	PWB7	0.813 ***			
	PWB8	0.803 ***			
	PWB9	0.801 ***			
	PWB10	0.798 ***			

Note: α ^1^: Cronbach’s alpha, CR ^2^: composite reliability, AVE ^3^: average variance extracted, ***: *p* < 0.001.

**Table 3:** Descriptive statistics and Fornell–Larcker method for discriminant validity.

Construct	Mean	Standard Deviation	1	2	3	4
1- Transformational leadership	4.23	0.893	0.864 ^a^			
2- Job satisfaction	4.18	0.851	0.710 ***^b^	0.822 ^a^		
3- Employee engagement	4.27	0.910	0.750 ***^b^	0.699 ***^b^	0.902 ^a^	
4- Psychological well-being	4.38	0.645	0.754 ***^b^	0.756 ***^b^	0.880 ***^b^	0.794 ^a^

Note: ^a^: the square root of AVE construct. ***^b^: latent variable correlation (***: *p* < 0.001).

**Table 4:** Discriminant validity by indicator cross-loading and collinearity statistics (VIF).

Indicators	TLS	JS	EEG	PWB	VIF
TLS1	0.939	0.557	0.605	0.839	1.826
TLS2	0.895	0.500	0.534	0.795	1.815
TLS3	0.835	0.475	0.527	0.735	1.736
TLS4	0.923	0.530	0.544	0.823	1.544
TLS5	0.910	0.557	0.545	0.810	1.852
TLS6	0.706	0.492	0.548	0.606	1.377
TLS7	0.817	0.643	0.690	0.717	1.603
JS1	0.654	0.807	0.658	0.595	1.839
JS2	0.543	0.797	0.608	0.587	1.693
JS3	0.607	0.882	0.662	0.610	1.912
JS4	0.590	0.855	0.623	0.595	1.872
JS5	0.456	0.761	0.543	0.522	2.940
EEG1	0.694	0.426	0.858	0.489	2.607
EEG2	0.687	0.423	0.940	0.492	1.768
EEG3	0.672	0.444	0.911	0.484	1.706
EEG4	0.617	0.446	0.705	0.412	1.455
EEG5	0.705	0.436	0.960	0.496	1.800
EEG6	0.644	0.557	0.952	0.627	1.816
EEG7	0.619	0.428	0.805	0.447	2.001
EEG8	0.683	0.489	0.985	0.546	2.233
EEG9	0.705	0.521	0.960	0.482	1.986
PWB1	0.644	0.557	0.687	0.777	2.147
PWB2	0.564	0.484	0.632	0.722	2.132
PWB3	0.619	0.428	0.663	0.753	1.842
PWB4	0.683	0.489	0.709	0.799	2.711
PWB5	0.596	0.654	0.737	0.827	2.479
PWB6	0.568	0.601	0.756	0.846	1.803
PWB7	0.607	0.595	0.723	0.813	2.943
PWB8	0.611	0.480	0.713	0.803	2.747
PWB9	0.705	0.521	0.711	0.801	1.684
PWB10	0.588	0.503	0.711	0.798	1.911

Note: TLS: transformational leadership, JS: job satisfaction, EEG: employee engagement, PWB: psychological well-being, VIF: variance inflation factor.

**Table 5:** Structural model fit.

Construct	R-Square	R-Square Adjusted	Q^2^ Predict	f^2^		
				EEG	JS	PWB
EEG	0.563	0.562	0.558		0.146	0.832
JS	0.567	0.565	0.498			0.130
PWB	0.818	0.817	0.565			
TLS				1.287	0.182	0.028

Note: TLS: transformational leadership, JS: job satisfaction, EEG: employee engagement, PWB: psychological well-being.

**Table 6:** Structural Parameter Estimates.

Hypothesized Path	Original Sample (O)	Sample Mean (M)	Standard Deviation (STDEV)	T Statistics	*p* Values	Confidence Intervals		Result
						2.5%	97.5%	
Direct Paths								
H_1_: TLS -> PWB	0.117	0.115	0.037	3.117 **	0.002	0.045	0.191	Accepted
H_2_: TLS -> EEG	0.750	0.751	0.024	31.919 ***	0.000	0.699	0.791	Accepted
H_3_: EEG -> PWB	0.630	0.631	0.034	18.370 ***	0.000	0.560	0.695	Accepted
H_5_: TLS -> JS	0.425	0.424	0.052	8.116 ***	0.000	0.318	0.524	Accepted
H_6_: JS -> PWB	0.233	0.233	0.039	6.004 ***	0.000	0.158	0.308	Accepted
H_8_: EEG -> JS	0.380	0.382	0.051	7.393 ***	0.000	0.277	0.481	Accepted
Indirect Paths								
H_4_: TLS -> EEG -> PWB	0.472	0.474	0.029	16.561 ***	0.000	0.419	0.532	Accepted
H_7_: TLS -> JS -> PWB	0.099	0.099	0.023	4.401 ***	0.000	0.059	0.146	Accepted
H_9_: TLS -> EEG -> JS -> PWB	0.067	0.067	0.014	4.623 ***	0.000	0.041	0.099	Accepted

Note: TLS: transformational leadership, JS: job satisfaction, EEG: employee engagement, PWB: psychological well-being, ***: *p* < 0.001, **: *p* < 0.01.

## Data Availability

The data presented in this study are available on request from the corresponding authors.

## Appendix A

**Table A1:** Study constructs and their related items.

Construct	Items	Statement
Transformational leadership (TLS)	TLS1	Your manager communicates a clear and positive vision of the future.
	TLS2	Your manager treats staff as individuals, supports and encourages their development
	TLS3	Your manager gives encouragement and recognition to staff
	TLS4	Your manager fosters trust, involvement, and cooperation among team members
	TLS5	Your manager encourages thinking about problems in new ways and questions assumptions
	TLS6	Your manager is clear about his/her values and practices what he/she preaches
	TLS7	Your manager instils pride and respect in others and inspires you by being highly competent
Employee engagement (EEG)	EEG1	I am willing to really push myself to reach challenging work goals.
	EEG2	I am prepared to fully devote myself to performing my job duties.
	EEG3	I get excited thinking about new ways to do my job more effectively.
	EEG4	I am enthusiastic about providing a high-quality product or service.
	EEG5	I am always willing to“go the extra mile”in order to do my job well.
	EEG6	Trying to constantly improve my job performance is very important to me.
	EEG7	My job is a source of personal pride.
	EEG8	I am determined to be complete and thorough in all my job duties.
	EEG9	I am ready to put my heart and soul into my work
Job satisfaction (JS)	JS1	How do you feel about your job?
	JS2	How do you feel about the people you work with (your co-workers)?
	JS3	How do you feel about the work you do on your job (the work itself)?
	JS4	What is it like where you work (the physical surrounding, the hours, the amount of work you are asked to do)?
	JS5	How do you feel about what you are available for doing your job (i.e., equipment, information, good supervision and so on)?
Psychological well-being (PWB)	PWB1	I easily adapt to day-to-day changes of my life and manage my responsibilities well.
	PWB2	I care for things that are important to me, not what is important to others.
	PWB3	I feel I am a sensible person.
	PWB4	I am flexible
	PWB5	I understand my expectation of myself.
	PWB6	I feel I am capable of decision-making
	PWB7	I feel psychologically comfortable and able to cope with the demands of daily life
	PWB8	I believe that I have a purpose and direction in life.
	PWB9	I think life is a continuous process of learning
	PWB10	I am a confident person.
